# Supplementary material for: Exploring the Impact of Efavirenz on Aflatoxin B1 Metabolism: Insights from a Physiologically Based Pharmacokinetic Model and a Human Liver Microsome Study
Source: Toxins (Basel). 2024 Jun 4;16(6):259. doi: 10.3390/toxins16060259 (PMC11209285; doi:10.3390/toxins16060259)
Supplement: Supplementary file 1 [file toxins-16-00259-s001.zip › toxins-2979484-supplementary.pdf]

toxins

Exploring the Impact of Efavirenz on Aflatoxin B1 Metabolism: Insights from a Physiologically Based Pharmacokinetic Model and a Human Liver Microsome Study

Orphélie Lootens, Marthe De Boevre, Elke Gasthuys, Sarah De Saeger, Jan Van Bocxlaer and An Vermeulen

Table S1. The gradient program used for the liquid chromatography-tandem mass spectrometry (LC-MS/MS) at a constant flow rate of 0.250 mL/min**.**

time (min)	solvent A (%)	solvent B (%)
initial	60	40
2.5	60	40
3.5	55	45
8.5	10	90
10	60	40
12	60	40

* Solvent A = H_2O/MeOH/acetic acid (94/5/1, v/v/v) + 5 mM ammonium acetate.

Solvent B = MeOH/ H_2O/acetic acid (97/2/1, v/v/v) + 5 mM ammonium acetate.

Table S2. Multiple reaction monitoring transitions and mass spectrometry settings for aflatoxin B1 (AFB1), aflatoxicol (AFL), aflatoxin M1 (AFM1), aflatoxin Q1 (AFQ1), zearalanone (ZAN), used as internal standard, midazolam (MDZ), 1′-hydroxymidazolam (1′-OH-MDZ) and chlorpropamide (CHL), used as internal standard.

Channel	Retention time (min)	Precursor ion (Da)	Product ion (Da)	Dwell (s)	Cone (V)	Collision (eV)
			**AFB1**			
1	5.07	313.0	241.1	0.033	65.00	32.00
2	5.07	313.0	270.1	0.033	70.00	35.00
			**AFL**			
1	6.89	296.9	226.0	0.032	45	25
2	6.89	296.9	269.0	0.032	45	20
			**AFM1**			
1	2.96	329.05	259.1	0.044	30.00	25.00
2	2.96	329.05	273.1	0.044	30.00	22.00
			**AFQ1**			
1	2.84	329.1	177.0	0.038	70.00	20.00
2	2.84	329.1	283.1	0.038	70.00	18.00
3	2.84	329.1	311.1	0.038	70.00	28.00
			**ZAN (IS)**			
1	9.13	321.0	189.1	0.033	35.00	22.00
2	9.13	321.0	303.3	0.033	35.00	14.00
			**MDZ**			
1	5.16	326.0	223.0	0.033	50.00	30.00
2	5.16	326.0	249.0	0.033	50.00	30.00
3	5.16	326.0	291.0	0.033	50.00	20.00
			**1′-OH-MDZ**			
1	6.95	342.0	203.0	0.033	25.00	22.00
2	6.95	342.0	289.0	0.033	25.00	22.00
3	6.95	342.0	324.0	0.033	25.00	20.00
			**CHL (IS)**			
1	6.33	277.0	111.0	0.033	30.00	25.00
2	6.33	277.0	175.0	0.033	30.00	12.00
3	6.33	277.0	192.0	0.033	30.00	7.00

*IS = internal standard.
